# Communion supports dignity for older adults with serious cancer: Quantitative findings from dignity therapy intervention

**DOI:** 10.1017/S1478951524001329

**Published:** 2024-10-23

**Authors:** Mary Kate Koch, Sophia Maggiore, Carma L. Bylund, Harvey Max Chochinov, Sheri Kittlelson, Diana Wilkie, Susan Bluck

**Affiliations:** 1 Department of Psychology, University of Florida, Gainesville, FL, USA; 2 University of Manitoba, Canada

**Keywords:** Dignity therapy, narration, palliative care, terminal care, psychosocial intervention

## Abstract

**Objectives.:**

Patient dignity is a key concern during end-of-life care. Dignity Therapy is a person-centered intervention that has been found to support patient dignity interviews focused on narrating patients’ life stories and legacies. However, mechanisms that may affect utility of the Dignity Therapy have been little studied. In this study, we evaluate whether the extent to which patients are more communal in their interviews acts as a mechanism for increased patient dignity.

**Methods.:**

We analyzed the written transcripts from Dignity Therapy interviews with 203 patients with cancer over the age of 55 receiving outpatient palliative care (*M* = 65.80 years; *SD* = 7.45 years, Range = 55–88 years; 66% women). Interviews followed core questions asking patients about their life story and legacy. We used content-coding to evaluate the level of communion narrated in each interview, and mediation analyses to determine whether communion affected dignity impact.

**Results.:**

Mediation analyses indicated that the extent to which patients narrated communion in their interview had a significant direct effect on post-test Dignity Impact. Communion partially mediated the effect of pre-test on post-test Dignity Impact. For both the life story and legacy segments of the session, narrating communion had a direct effect on post-test Dignity Impact.

**Significance of results.:**

Narrating communion serves as a mechanism for enhancing patient dignity during Dignity Therapy. Providers may consider explicitly guiding patients to engage in, elaborate on, communal narration to enhance therapeutic utility. In addition, encouraging patients with advanced illness to positively reflect on relationships in life may improve patient dignity outcomes in palliative and end-of-life care.

## Introduction

Psychosocial well-being is a key component of quality of life during palliative care (Madan 1992). Maintaining dignity is difficult for patients if they are experiencing existential and psychological burdens (Chochinov 2006). Dignity Therapy is a brief, person-centered psychotherapy designed to foster dignity in patients with advanced disease (Chochinov 2002). The intervention involves providing a forum to reflect on one’s life story and consider the personal legacy they wish to leave loved ones. Myriad research indicates Dignity Therapy is effective for enhancing the dignity of individuals suffering from advanced diseases, including cancer (Fitchett et al. 2015). However, some studies have found mixed results (Fitchett et al. 2015; Xiao et al. 2019). As Dignity Therapy implementation spreads internationally, identifying mechanisms underlying its effectiveness is critical (Xiao et al. 2019) – if we can identify these mechanisms, then we can work to bolster them in the implementation. The present study focused on examining one potential mechanism, the extent of communion participants narrate when talking about their life and legacy during the Dignity Therapy session.

Dignity Therapy is a multidimensional intervention centered on reminiscence and generativity, and grounded in a life story-legacy interview. The content of patients’ narrative responses may be one factor central to effectiveness. Providers guide patients in 2 contexts of narration: telling their life story and considering the legacy they want to leave (e.g., hopes, dreams, instructions they have for loved ones). That is, patients respond to core questions cueing them to look back on the life lived (i.e., life story) and look ahead to when they will be gone (i.e., legacy) (Chochinov et al. 2005). This process is based on the notion that patients’ sense of dignity will increase post-therapy: narration should foster role preservation, encourage a sense of self-continuity, facilitate a reflection on integrity across the lived life, allow expression of generativity toward important others, and a sense one’s influence will transcend death through others (Chochinov et al. 2002).

While all patients respond with a life story-legacy narrative during Dignity Therapy, how they tell these stories varies (McAdams 2001b; Mroz et al. 2020). Telling personal stories that are rich, organized, and affect-laden has been linked to greater psychosocial outcomes while telling skeletal, fractured, and emotionally neutral stories, even if factually correct, has not (Bauer and Park 2010; Neimeyer 2019). In particular, communion may be a critical component in telling a rich narrative during Dignity Therapy. Based in life story research, communion is defined as the extent to which individuals use a lens of love and care when reflecting on their life story and considering their legacy (McAdams et al. 1996). While individuals may also benefit from sharing difficult or painful sentiments regarding others, communion focuses specifically on these positive elements. Greater communion in life story and legacy narration is linked with an array of positive, health-related outcomes, including greater life satisfaction, greater well-being, and better mental health (Adler et al. 2017; Grossbaum and Bates 2002; Grysman 2022). Accordingly, we hypothesize that greater communion narration during the life story-legacy interview of Dignity Therapy is one mechanism of increased patient dignity post-intervention.

Recalling one’s life story and generating a legacy, though both part of Dignity Therapy, are different psychosocial tasks for the patient. Narrating greater communion during the life story may support patient dignity through guided reflection on their past lived experience of having caring, warm relationships (McAdams et al. 1996). In contrast, communal legacy narration involves looking into one’s future to create a generative, caring narrative regarding wishes and goals for loved ones that will be left behind after death (McAdams et al. 1997). Note that these constructs do not simply capture individuals who had more loving, warm relationships during their life. Rather, communal narration reflects an individual difference in how patients select and communicate memories. With the goal of increasing Dignity Therapy utility, it is useful to identify whether dignity outcomes are impacted by communion during either or both the life story and legacy portion of Dignity Therapy to target improvements in how providers may facilitate and support patients.

## The current study

This study has 3 central aims. First, the extent of communion expressed in the Dignity Therapy session is evaluated as a mechanism (i.e., mediator) for facilitating greater patient dignity from pre- to post-test. Second, the effects of communion narration during the life story and during the legacy portions of Dignity Therapy are examined separately to determine whether one part of the Dignity Therapy session is key for increasing patient dignity. Finally, exemplars of how communion manifests in Dignity Therapy are provided to elaborate on communal themes that support individual patient dignity.

## Methods

### Design

Data for this study was drawn from a 3-arm, pre- and post-test, randomized, controlled 4-step, stepped-wedge study examining the use of Dignity Therapy to improve seriously ill cancer patients’ sense of dignity (Kittelson et al. 2019). The present study conducted secondary data analysis on data from participants who completed Dignity Therapy using content coding and mediation analyses.

### Setting

Recruitment occurred in 6 large U.S. medical centers.

### Recruitment

Eligibility criteria included: (1) diagnosed with cancer, (2) receiving outpatient palliative care, (3) 55 years or older, (4) speaks and reads English, and (4) able to complete the study.

### Sample

Participants were 203 older adults (*M* = 65.80 years; *SD* = 7.45 years, Range = 55–88 years; 66.01% women). They self-identified their race/ethnicity as 77.94% White, 11.76% Black or African-American, 7.84% Hispanic or Latino, .49% American Indian or Alaska Native; .49% Asian, .49% Native Hawaiian or other Pacific Islander, and .98% declined. The majority (82.84%) had completed education post-high school and about half (48.53%) had a college degree.

### Data collection

Participants completed self-report measures about sense of dignity, symptom severity, and demographics. They then engaged in a Dignity Therapy interview with a trained provider following a standardized interview protocol framed by 9 core questions. Four questions focused on telling one’s life story (e.g., “Tell me a little about your life history; particularly the parts that you either remember most or think are the most important?”) and 5 focused on leaving a legacy (e.g., “Are there words or instructions that you would like to offer your family to help prepare them for the future?”) (4, 5). About 3–5 weeks later, participants completed a post-test assessing impact on their sense of dignity. Dignity Therapy sessions were audio-recorded, transcribed, and de-identified for narrative coding analysis. Average duration of sessions was 48.70 minutes (*SD* = 13.10). Patients were compensated $150 for completion. All data were collected between 2019 and 2022.

### Ethics and consent

The Institutional Review Board at each of the 6 sites approved the study (https://clinicaltrials.gov: NCT03209440). The University of Florida Institutional Review Board (RB201601190, “Dignity Therapy for Older Cancer Patients: Identifying Mechanisms and Moderators”) approved all research activities. All participants provided written informed consent.

## Measures

### Dignity impact

The major variable of interest was patient dignity. The Dignity Impact Scale is a 7-item scale assessing impact of care on patients’ current sense of dignity and psychological well-being (Chochinov et al. 2011). Patients respond on 5-point scales from “strongly disagree” to “strongly agree” to statements such as “The care I received during the past month has given me a heightened sense of purpose.” Other items tap into patient dignity through questions about whether recent care helped resolve unfinished business, made life feel more meaningful, lessened sense of depression and helped with family connection. Sum scores ranged from 7 to 35 (*M* = 24.31, *SD* = 4.38) at pre-test and 13 to 35 (*M* = 26.21, *SD* = 4.57) at post-test. Cronbach’s α was .79 at pre- and .84 at post-test.

### Communion in Dignity Therapy narratives

Communion, defined as expression of loving, caring or closeness with others, was coded using narrative analysis (McAdams et al. 1996). We adopted a modified version of a well-established codebook for assessing communion in open-ended narrative data (McAdams 2001a). We employed best practices for narrative analysis (Adler et al. 2017; Bluck et al. 2022). Two coders were rigorously trained on pilot narratives and achieved strong inter-rater reliability (κ = .85). Communion was coded as present when utterances referred to: love and friendship toward others, caring and helping others, and feelings of unity and togetherness. Transcripts were divided into idea units that were coded with a “1” if the subtheme was present and a “0” if the subtheme was absent. Accordingly, each idea unit could receive a sum communion score from 0 (no communion present) to 3 (love and friendship, caring and help, and unity togetherness all present). Narratives were double-coded for additional rigor. Remaining discrepancies were resolved through discussion. Coder drift was mitigated through regular meetings. Across the whole Dignity Therapy session, mean number of instances of communion ranged from 0 to 26 (*M* = 8.29, *SD* = 4.66). Communion ranged from 0 to 17 instances (*M* = 5.12, *SD* = 3.24) within the life story portion and 0 to 18 (*M* = 3.16, *SD* = 2.71) in the legacy portion.

### Symptom severity

We assessed symptom severity to adjust for patients’ mental health and physical functioning. Symptom severity was measured with the 10-item Edmonton Symptom Assessment System assessing pain, tiredness, nausea, depression, anxiety, drowsiness, appetite, well-being, shortness of breath, and other symptoms (Watanabe et al. 2012). Items were measured on a Likert scale where 0 = no presence of symptom and 10 = worst possible experience of symptom. Sum scores ranged from 0 to 77 (*M* = 26.90, *SD* = 15.02) at pre-test. Scale reliability was good (α = .77).

### Data analyses

We addressed the first aim with a model examining communion as a mediator of improvement in Dignity Impact from pre- to post-test. For Aim 2, we conducted an additional model assessing if mediation effects differed by whether communion occurred during the life story or the legacy portion of Dignity Therapy. Given that life story focuses on reminiscence and legacy centers on future generativity, we anticipated that communion may function differently during these sections. Race, education level, and symptom severity were covariates in all models. Complete data was used. Models were fit in M*Plus* 7.4 (Muthén and Muthén 1998). Model fit is considered good if the comparative fit index (CFI) is greater than or equal to .95, root mean square error of approximation (RMSEA) is less than or equal to .06, and standardized root mean square residual (SRMR) is less than or equal to .05 (Kline 2005). To address the third aim, we extracted exemplars from patients’ narratives.

## Results

### Aim 1. To evaluate whether extent of communion expressed in the Dignity Therapy session acts as a mechanism for increased dignity from pre- to post-test

**Figure 1.**
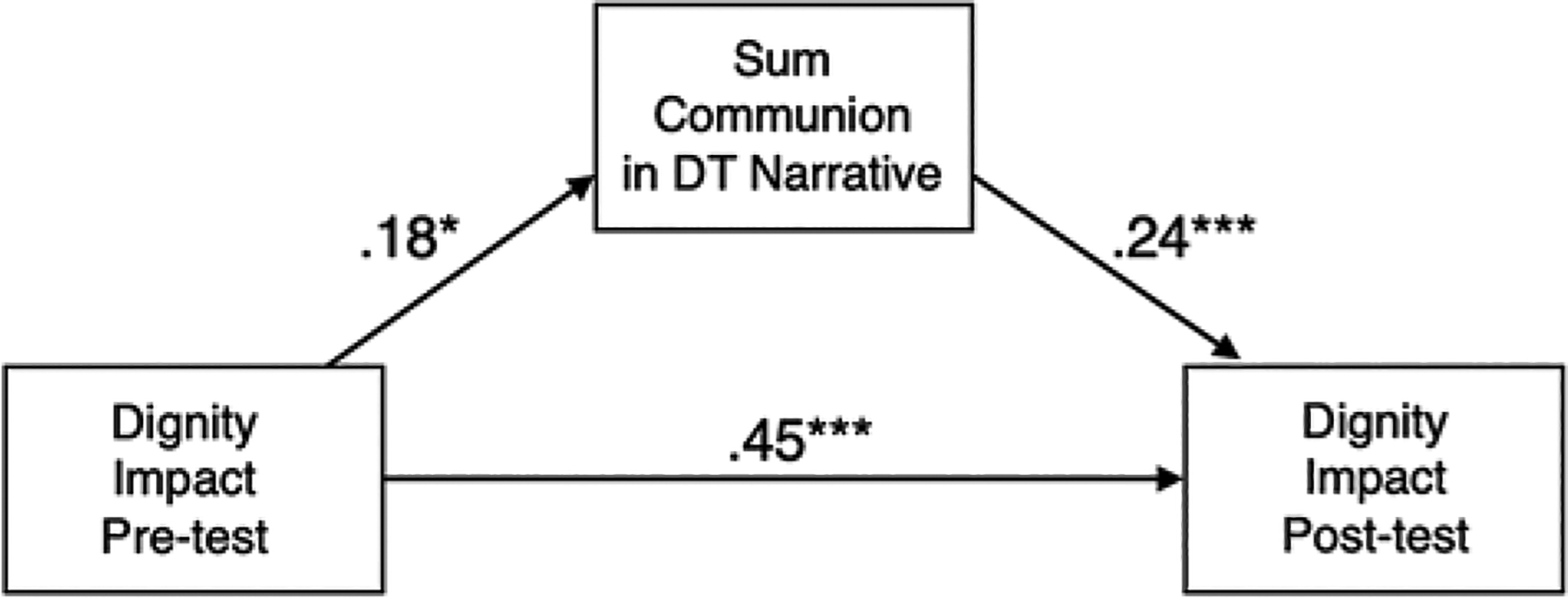
Sum communion mediation model. *Note*: Standardized estimates are presented. Covariates are not presented in the figure for ease of interpretation but are described in text. Covariates include race, education, and symptom severity. Model fit was good: CFI = .99, RMSEA = .03 [.00, .13], SRMR = .03. * = *p* < .05. ** = *p* < .01. *** = *p* < .001.

Results from the first communion mediation model are shown in Fig. 1. The relation of Dignity Impact from pre- to post-test was partially explained by an indirect effect through extent of communion narration (*B* = .05, *p* < .05), indicating mediation. Further, narrating a greater extent of communion in their Dignity Therapy session was significantly and directly related to more positive Dignity Impact at post-test (*B* = .24, *p* < .001). Addition of covariates did not affect the pattern of findings.

### Aim 2. To evaluate whether this effect differs for the life story and the legacy portions of patients’ narratives

**Figure 2.**
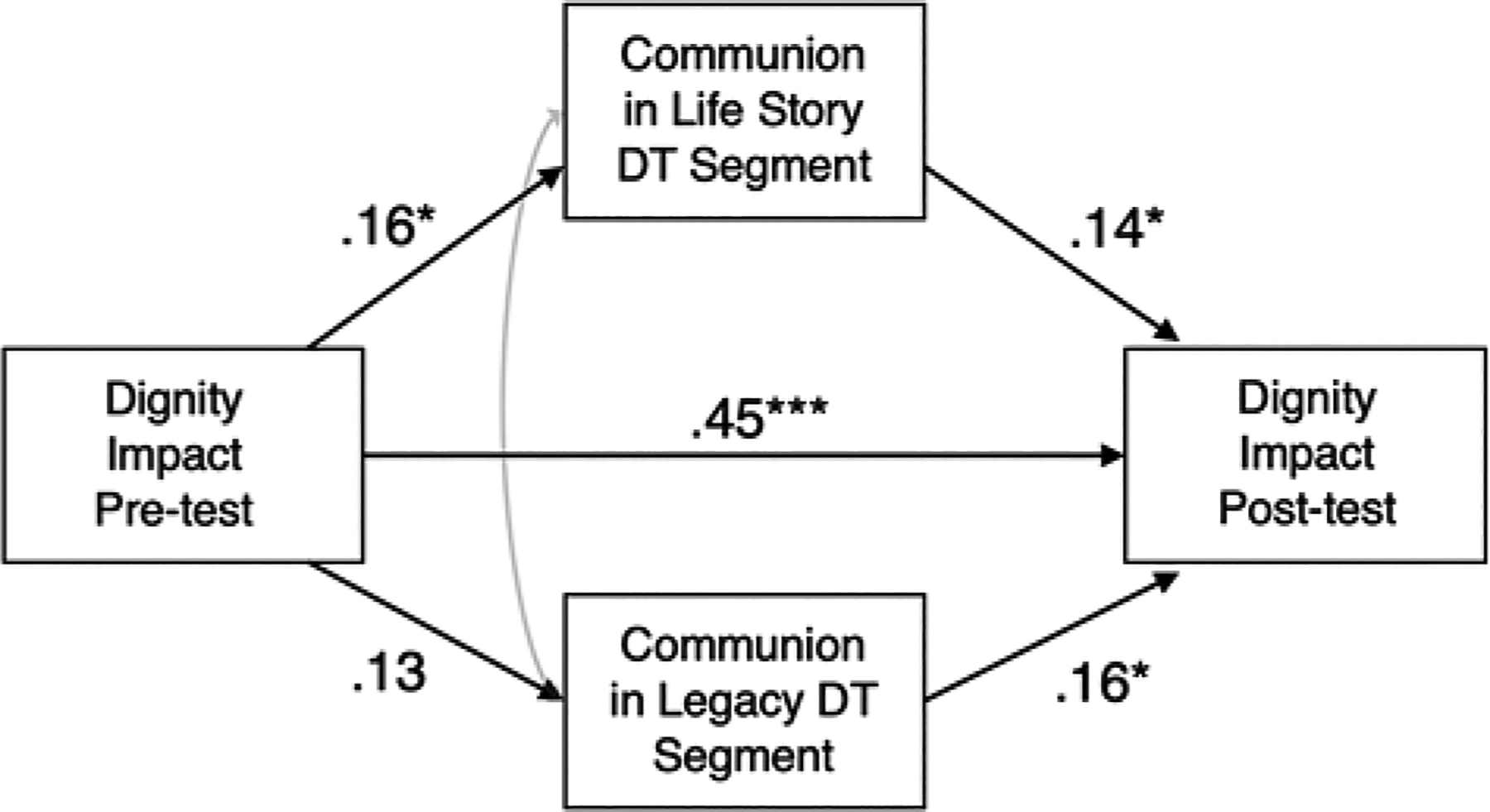
Communion in life story and legacy DT segments mediation model. *Note*: Standardized estimates are presented. Covariates are not presented in the figure for ease of interpretation but are described in text. Covariates include race, education, and symptom severity. Model fit was good: CFI = 1.00, RMSEA = .00 [.00, .09], SRMR = .03. * = *p* < .05. ** = *p* < .01. *** = *p* < .001.

Greater communion in both the life story (*B* = .14, *p* < .05) and legacy (*B* = .16, *p* < .05) segments of Dignity Therapy had significant direct effects on post-test Dignity Impact (see Fig. 2). However, neither life story communion (*B* = .02, *p* = .12) nor legacy (*B* = .02, *p* = .14) communion individually contributed indirect effects to relations between pre-test Dignity Impact and post-test Dignity Impact. That is, patients’ expression of communion could occur anywhere in the Dignity Therapy session and still support increased patient dignity. Addition of covariates did not affect the pattern of findings.

### Aim 3. To provide exemplars of how communion manifests in Dignity Therapy sessions

Patients often talked about typical life events (e.g., work-life, children). However, they differed in how they narrated events. Patients high in communion described life experiences emphasizing love and caring. For example, one high communion participant described first meeting her spouse, including the warm connection they quickly shared (Table 1). In contrast, a low communion participant described a similar event but did so without a communal focus. Patients who narrated more than 13 instances of communion were considered exemplars of high communion whereas those with less than 4 instances were considered exemplars of low communion. It is the events that occur in one’s life but also the ways in which individuals tell their stories and legacy that is critical to patient dignity outcomes.

## Discussion

Patient dignity is a paramount concern in end-of-life care, particularly for individuals grappling with serious and terminal illnesses. Dignity Therapy has emerged as an effective intervention to address this concern with international implementation success. Patients are offered a person-centered approach that allows them to narrate their life stories and legacies. While previous research has demonstrated the utility of Dignity Therapy in enhancing patient dignity, there remains a need to identify mechanisms underlying its effectiveness. Present quantitative findings provide compelling evidence that the extent of communion expressed by patients during Dignity Therapy is a significant mechanism for enhancing patient dignity between pre- and post-intervention. In addition, the effect of communion was not limited to a specific segment of the Dignity Therapy session. This underscores the importance of fostering communion throughout the entirety of the therapeutic process, rather than focusing solely on 1 aspect of the patient’s narrative.

Quantitative findings suggest that warm, loving relations when facing advanced cancer may support patient dignity during Dignity Therapy interventions. These trends may also be expanded to other aspects of clinical care. For instance, narrative exemplars high-lighted the significance of interpersonal relationships and emotional connections in supporting patient dignity, reinforcing the importance of a person-centered approach in end-of-life care. Accordingly, the encouragement of communication rich responses or narratives could apply to other healthcare settings outside of Dignity Therapy, such as palliative and psycho-oncological care. Although time may be more limited in other care settings compared to Dignity Therapy, incorporating a brief life review question may be beneficial for patient dignity (e.g., What was your most memorable role?). Specific to Dignity Therapy, providers may be trained to encourage patients in communal expression during sessions to enhance patient dignity. However, this should be attuned to the patient as individuals wanting to express remorse or regret may not be interested or benefitted by such redirection.

The present study raises several intriguing questions for further investigation that may support insight into providing quality care to seriously ill patients. Given present results benefiting psychosocial outcomes, future research should identify when Dignity Therapy may also relieve physical symptoms. This may contribute to end-of-life healthcare utilization through improving patient outcomes while limiting unwanted burdensome care. In addition, Dignity Therapy providers may test whether using questions that reframe negative emotions (e.g., “What was learned from that challenging experience that helped the relationship grow in the future?”) also helps patients experience greater dignity outcomes.

Limitations of the present study include that the sample predominantly consisted of older adult patients with cancer receiving outpatient palliative care, limiting the generalizability of the findings to other populations. Further, the present sample was American and predominately White. While effects held even when accounting for patients’ race, education level, and symptom severity, future research is needed to understand whether Dignity Therapy performs optimally under different conditions for patients from a broader range of racial and cultural backgrounds. Finally, the present data did not include relationship quality measures, which may be important to include as a covariate when considering the effects of communion in life story and legacy narration.

A strength of the present study was the use of the life story and legacy narratives that are produced by patients during Dignity Therapy sessions. By examining these interviews quantitatively, we were able to identify how mechanisms that occur during sessions may impact patient dignity outcomes. This is important because prior work has not examined the narrative processes that may help explain why Dignity Therapy is effective for patient dignity.

## Conclusions

This study contributes to our understanding of the mechanisms underlying the effectiveness of Dignity Therapy in enhancing patient dignity. By highlighting the role of communion in patient narratives during therapy sessions, we provide valuable insights for improving psychosocial interventions in end-of-life care. In addition, Dignity Therapy is a therapeutic approach that can be applied to palliative care in the shared pursuit of understanding and supporting the dying person. Palliative care teams may draw similar insights from present findings to help further understand the dynamics of fostering patient dignity during clinical practice. Future research should continue to explore the complex interplay between narrative elements and patient outcomes to further optimize the delivery of person-centered care for individuals facing advanced illness.

## Figures and Tables

**Table 1.** Examples of high and low communion narration during dignity therapy

Participant	Life story segment	Participant	Legacy segment
*High communion participants*			
Female, White, 65 years old	*We [husband and narrator] just connected. It was like we didn’t even have to talk. It was like a heart-to-heart thing. We could just look at each other and know that we belong to each other*.	Female, White, 56 years old	*I don’t want him to feel that pain. I don’t ever want him [father] to feel that pain ‘cause I saw the pain that he felt when his wife died, when my mother died, and he’s still not over it*.
Female, White, 69 years old	*Basically, every single job that I took had to do with people in need. I’ve worked at the hospital helping people get Medicaid so they can pay their hospital bill. I’ve worked with foster children. I’ve worked with drug addicts. I’ve worked with inmates. I’ve worked with abused women*.	Female, African- American, 64 years old	*She [niece] says all the time, “But I’m just like you, Auntie.” It’s so far from the truth but I just want her to know that [Auntie] always loved her. I always will and I’ll always be looking on her, no matter where I’m looking from*.
Male, White, 68 years old	*She [mother] just held me and said, “hey, we’ll work through this together.” I’ve stayed here at the house and then without her, I don’t think I woulda kicked drugs tell ya the truth. It’s just, I don’t know if she just, I don’t know*.	Male, White, 62 years old	*Running for example, I never really loved to run, but I liked to run because it was good for me, and I didn’t want to die young. [Laughter] I wanted to be around for my children and grandchildren a long time. I did things like that because I wanted to be the best I could for them and be around for a long time*.
*Low communion participants*			
Male, White, 59 years old	*I met their mother up there in Maine, and that was another story too. My mother, my uncle couldn’t stand her, so she went with me to-’cause she was born and raised in Maine, but she was southern Maine, [town]. When I left, she went with me. That’s when one of my daughters were born*.	Male, White, 78 years old	*Well, the biggest thing for them to know, and they will know it because it is intuitive, and in the blood, they got to know, you got to get up and show up and that is the biggest thing to success. If you can get up and put your clothes on and go to work, go to school, get up and show up. If you can do those two things, 98% success right there*.

*Note*: Narrative exemplars from participants who showed high levels (more than 13 occurrences) and low levels (less than 4 occurences) of communion throughout their narratives. Exemplars are included from legacy and life story sections of the transcript.
